# Suprascapular nerve entrapment syndrome caused by a spinoglenoid notch cyst with a concomitant giant lipoma: a case report

**DOI:** 10.3389/fsurg.2025.1737191

**Published:** 2026-01-12

**Authors:** Zaichao Ma, Mengxue Guan, Maimaitiyibubaji Abudukadier, Xiaoping Han, Tao Huang, Zengqiang Yang, Biao Li, Yong Cui

**Affiliations:** 1 Department of Orthopedics, The Fifth Affiliated Hospital of Xinjiang Medical University, Urumqi, Xinjiang, China; 2 Department of Neurosurgery, The First Affiliated Hospital of Xinjiang Medical University, Urumqi, Xinjiang, China

**Keywords:** cyst, fatty lump, nerve, spinoglenoid notch, suprascapular nerve entrapment

## Abstract

**Background:**

Suprascapular nerve entrapment is a cause of shoulder pain and dysfunction, often complicated by symptomatic overlap with other shoulder pathologies. Entrapment most commonly occurs at two anatomical constrictions: the suprascapular notch and the spinoglenoid notch. Compression of the nerve's inferior branch at the spinoglenoid notch by a paralabral cyst, leading to isolated infraspinatus weakness and atrophy, is a relatively common pattern. Diagnosis relies on a detailed physical examination, multimodal imaging evaluation including MRI and ultrasound, and confirmation by electromyography. For patients who do not respond to conservative management or who have definitive space-occupying compression, surgical decompression is an effective treatment option.

**Case presentation:**

This is the case of a 27-year-old man presenting with progressive right shoulder weakness and pain over just one month, already demonstrating isolated infraspinatus atrophy. Imaging revealed the etiology to be a paralabral cyst that, notably, occupied both the suprascapular and spinoglenoid notches, creating a “double-crush” compression on the suprascapular nerve. This case clearly illustrates how a strategically located space-occupying lesion can lead to rapid and characteristic neurologic deficit, even within a short clinical course.

**Conclusion:**

This case clearly illustrates the classic presentation of an isolated spinoglenoid notch cyst causing suprascapular nerve compression, underscoring that this diagnosis must be considered in patients with isolated external rotation weakness even without a clear traumatic etiology, and highlighting that early recognition and systematic evaluation are key to successful management and neurological recovery.

## Background

Suprascapular nerve entrapment is a recognized cause of shoulder pain, accounting for approximately 2% of cases. Its diagnosis is frequently delayed or missed during initial assessment due to symptomatic overlap with other shoulder pathologies and the need for differentiation from cervical spine disorders (1, 2). This diagnostic challenge stems from its complex and multifactorial etiology, which includes traumatic causes such as scapular fractures and glenoid labral tears, static compression from anatomical variations like the suprascapular transverse ligament or the spinoglenoid ligament, and space-occupying lesions such as ganglion cysts and tumors (3). The suprascapular nerve originates from the upper trunk of the brachial plexus, traverses the suprascapular notch to innervate the supraspinatus muscle and provide sensory fibers to the shoulder joint capsule, and then its inferior branch passes through the spinoglenoid notch to supply the infraspinatus muscle. Entrapment most commonly occurs at either the suprascapular notch or the spinoglenoid notch, with the former being more frequent (2, 4–6). Compression of the inferior branch at the spinoglenoid notch by a ganglion cyst is relatively common, while concomitant compression by fatty tissue is less frequently observed (7). Classical classification systems primarily distinguish between entrapment at the suprascapular notch, affecting the main nerve trunk, and at the distal spinoglenoid notch, affecting only the infraspinatus branch, with significant differences in clinical presentation, imaging features, and treatment strategies between the two types (8). Recent work by Al-Redouan et al. has further elucidated the pathogenic mechanisms and clinical implications of this classification (9–11). The radiating pain from suprascapular nerve entrapment typically localizes around the lateral scapula and superior shoulder. Proximal brachial plexus pain also differs in localization from cervical radiculopathy and pain from distal terminal nerve branches. The nerve is most commonly compressed at two anatomical sites: the suprascapular notch and the spinoglenoid notch. Compression at the spinoglenoid notch typically results in infraspinatus weakness and atrophy. Clinically, patients present with posterior shoulder pain and functional limitations. Magnetic resonance imaging is the key modality for confirming the presence of a ganglion cyst and assessing the degree of nerve compression. Currently, ultrasound is an emerging and increasingly utilized diagnostic tool (12–14). Furthermore, nerve conduction velocity/electromyography studies are often integral to the overall diagnostic workup. For patients who do not respond to conservative management or who have a cyst definitively associated with nerve compression, surgical intervention is recommended—encompassing open or arthroscopic decompression, with or without cyst aspiration (15–18). This case presents an instance of suprascapular nerve entrapment where a cyst occupied the entire suprascapular nerve pathway, concurrently compressing both the suprascapular and spinoglenoid notches. The patient achieved a favorable recovery following arthroscopic decompression combined with open neurolysis.

## Case report

**Figure 1:**
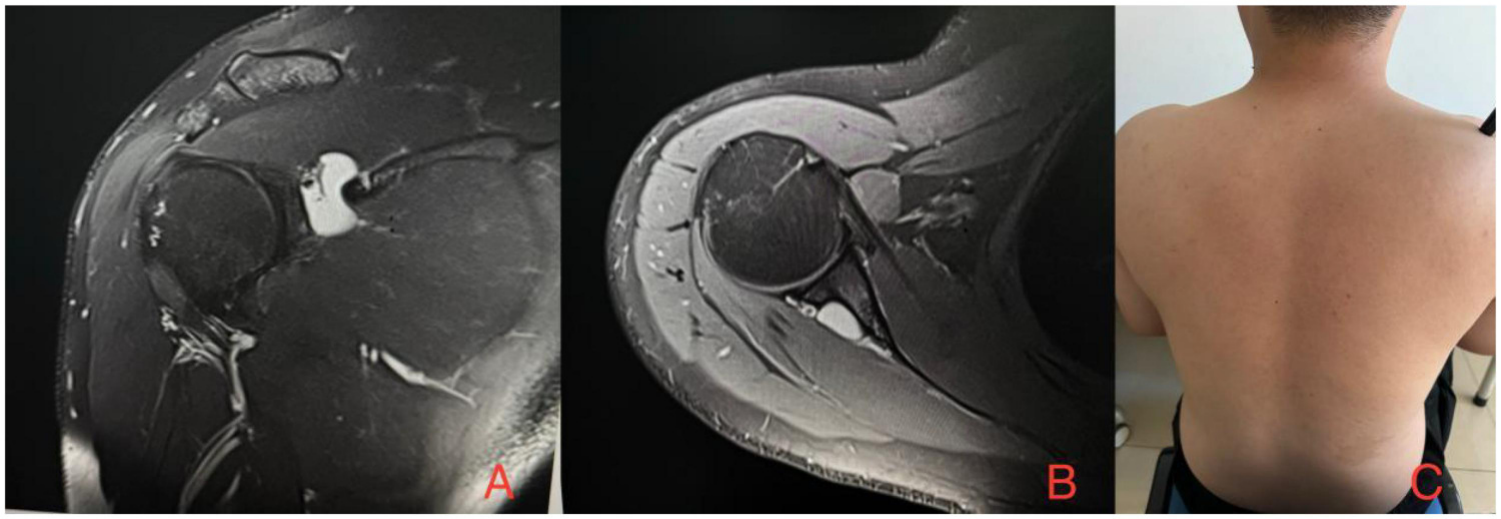
MRI of the right shoulder demonstrates a well-defined, unilocular abnormally hyperintense lesion (cyst) measuring approximately 2 cm × 2 cm at the spinoglenoid notch, which is in close proximity to the suprascapular nerve and adjacent vessels. **(A)**, Coronal view. **(B)**, Axial view. **(C)** Preoperative visual inspection revealed a mild depression in the right infraspinous fossa.

A 27-year-old male patient was admitted due to “right shoulder pain with limited movement for one month.” The patient is a young male with an insidious onset. One month ago, he developed persistent dull pain in the right shoulder joint without an obvious cause, which did not radiate elsewhere but was accompanied by progressively worsening limitation of shoulder flexion and abduction. The relationship between pain and activity was clear: it significantly worsened with activity and markedly improved with rest. Physical examination revealed mild hollowing of the infraspinatus fossa of the right shoulder, suggesting possible atrophy of the infraspinatus muscle. The muscle strength of external rotation and abduction of the right shoulder joint was both grade 3, significantly weaker than on the healthy side. Tenderness was present at the spinoglenoid notch, and positive findings on the abduction resistance test, cross-arm test, and scapular traction test all indicated involvement of the suprascapular nerve. Electromyography showed a significantly reduced amplitude of the compound muscle action potential (CMAP) in the infraspinatus muscle during suprascapular nerve motor conduction studies, while findings for the supraspinatus muscle were largely normal. Ultrasound revealed a well-defined, regularly shaped unilocular hypoechoic cystic structure within the deep muscle groups of the right shoulder. It clearly demonstrated signs of the nerve being elevated, displaced, or compressed and flattened by the cyst. MRI of the right shoulder displayed a unilocular abnormal high-signal focus at the spinoglenoid notch, with clear boundaries and close anatomical relationships to the suprascapular nerve and blood vessels. Based on imaging and anatomical correlations, this case clearly demonstrates a cyst occupying the entire suprascapular nerve pathway, compressing both the suprascapular notch and the spinoglenoid notch, resulting in suprascapular nerve entrapment (Figure 1).

Under general anesthesia, the patient was placed in the beach-chair position. Conventional shoulder arthroscopy was performed via posterior and anterosuperior portals to examine the glenohumeral joint and subacromial space. The glenoid labrum was intact without tear, though slightly frayed at the margin. The supraspinatus and infraspinatus tendons were intact. Adhesions were observed between the middle glenohumeral ligament and the subscapularis tendon. The fascia of the deltoid and trapezius was exposed, followed by blunt dissection of the trapezius fascia. The supraspinatus and infraspinatus muscles were separated, and the infraspinatus was mobilized. The posterior glenoid cavity and spinoglenoid notch were exposed, revealing a cyst measuring approximately 2 cm × 2 cm, with adipose tissue visible posteriorly. The suprascapular nerve was severely compressed and appeared pale locally.The cyst wall was resected with an arthroscopic shaver, and viscous, yellowish, jelly-like fluid was released. Subsequently, a lipoma-like mass was identified posteriorly and inferiorly to the cyst. Due to limited operating space, the arthroscope was withdrawn. The skin over the scapular spine was cleansed with alcohol, and an incision was made along the spine through the skin and subcutaneous tissue. Hemostasis was achieved with electrocautery. Part of the trapezius was incised, and blunt dissection was performed to expose the supraspinatus muscle. The posterior glenoid and spinoglenoid notch were subsequently exposed, revealing the lipoma-like adipose mass posterior to the original cyst attachment site, which was completely excised. Hemostatic material was applied to the wound and joint cavity for local hemostasis. The surgical site was irrigated with normal saline. The muscle and subcutaneous layers were closed in layers with absorbable sutures. The skin around the incision was disinfected again with alcohol and closed with intradermal sutures. The arthroscopic portal sites were closed with full-thickness sutures.

Postoperatively, the rehabilitation department was consulted to assist with functional exercise rehabilitation. Shoulder abduction and external rotation strength gradually recovered to grade 5. At the 6-month outpatient follow-up, shoulder motion had returned to normal with good stability. Follow-up MRI showed complete resolution of the spinoglenoid notch cyst and adipose tissue.

## Discussion

This case report describes the typical presentation of isolated shoulder external rotation weakness and infraspinatus atrophy due to compression of the inferior branch of the suprascapular nerve by a spinoglenoid notch cyst. Arthroscopic exploration revealed no associated labral injury, and the cyst contained adipose tissue, a finding that differs from the common mechanism of cyst formation secondary to labral tears (3). In contrast to most reports in the literature, where spinoglenoid cysts are associated with SLAP lesions or posterior labral tears, isolated cysts without an obvious labral injury, as seen in this case, have also been reported, with their etiology possibly involving synovial fluid extrusion through a labral recess or a microscopic channel (6). This finding suggests that cyst compression remains a possible etiology for suprascapular nerve entrapment even when conventional MRI does not show a definitive labral tear. The diagnostic and therapeutic process in this case adds several insights to the existing literature. Diagnostically, it reinforces the value of combining targeted physical examination—such as specific external rotation strength testing and inspection of the infraspinatus fossa—with multimodal imaging (MRI for definitive identification of the mass, ultrasound for dynamic nerve assessment, and electromyography to confirm nerve injury) (3, 19). This approach is particularly useful for early or atypical presentations, helping to avoid misdiagnosis as common cervical or shoulder soft-tissue disorders. Therapeutically, for cysts with no clear communication to the joint cavity and containing atypical components, arthroscopic exploration combined with limited open excision may be a reasonable option. This combined technique allows for assessment and management of possible concomitant intra-articular pathology while ensuring, via an open approach, complete excision of the cyst, particularly one with atypical contents, offering distinct advantages compared to isolated arthroscopic decompression or open surgery alone (20, 21). The patient in this case was treated with arthroscopic exploration and limited open excision, and at the 6-month follow-up, demonstrated significant symptomatic relief and excellent functional recovery. This aligns with literature reporting favorable neurological outcomes following early surgical decompression (17, 20, 21). Especially for patients with existing muscle atrophy but a relatively short symptom duration, timely relief of compression creates favorable conditions for neural recovery. A 2022 review also noted that the degree of post-decompression strength recovery is inversely correlated with the duration of preoperative nerve compression and the severity of muscle atrophy, underscoring the importance of early diagnosis and intervention (22). This study has certain limitations. First, it is a single-center case report, and caution should be exercised in generalizing the conclusions. Second, the patient's follow-up period was only 6 months; longer-term follow-up would be helpful to assess the final functional plateau and potential cyst recurrence.

## Conclusion

This report highlights the classic clinical and imaging features of an isolated compression neuropathy of the inferior branch of the suprascapular nerve by a spinoglenoid notch cyst. The case of a young man without a history of trauma, who presented with progressive isolated shoulder external rotation weakness and infraspinatus atrophy, serves as a well-localized diagnostic paradigm. The convergence of a characteristic clinical presentation, pathognomonic MRI findings, and confirmatory electrodiagnostic and ultrasonographic studies provides a complete chain of evidence. This case emphasizes that this diagnosis should be considered in patients presenting with shoulder pain and predominant external rotation weakness, even in the absence of a clear traumatic history or imaging evidence of a labral tear. Early evaluation through meticulous physical examination and multimodal imaging, followed by an individualized surgical approach tailored to the cyst's characteristics and the intra-articular findings, can lead to favorable neurological recovery. Further studies are needed to clarify the pathogenesis and optimal management strategy for such isolated cysts.

## Data Availability

The original contributions presented in the study are included in the article/Supplementary Material, further inquiries can be directed to the corresponding author.

## References

[1] Moen TC Babatunde OM Hsu SH Ahmad CS Levine WN. Suprascapular neuropathy: what does the literature show? J Shoulder Elbow Surg. (2012) 21(6):835–46. 10.1016/j.jse.2011.11.033 22445163

[2] Prenaud C Loubeyre J Soubeyrand M. Decompression of the suprascapular nerve at the suprascapular notch under combined arthroscopic and ultrasound guidance. Sci Rep. (2021) 11(1):18906. 10.1038/s41598-021-98463-1 34556759 PMC8460809

[3] Katsuura Y Hill AJ 4th Colón LF Dorizas JA. MRI diagnosis of suprascapular neuropathy using spinoglenoid notch distension. Radiol Med. (2019) 124(7):643–52. 10.1007/s11547-019-01005-z 30835024

[4] Boykin RE Friedman DJ Higgins LD Warner JJ. Suprascapular neuropathy. J Bone Joint Surg Am. (2010) 92(13):2348–64. 10.2106/JBJS.I.01743 20926731

[5] Ayik G Kolac UC Kaymakoglu M McFarland E Huri G. Dark side of the shoulder: suprascapular and axillary nerve compressions. Int Orthop. (2025) 49(5):1153–65. 10.1007/s00264-025-06465-9 40082300 PMC12003475

[6] Joo YB Lee WY Chung HJ. Suprascapular nerve entrapment caused by a large hematoma of the scapula: a case report. BMC Musculoskelet Disord. (2023) 24(1):589. 10.1186/s12891-023-06723-0 37468872 PMC10354896

[7] Knudsen ML Hibbard JC Nuckley DJ Braman JP. Anatomic landmarks for arthroscopic suprascapular nerve decompression. Knee Surg Sports Traumatol Arthrosc. (2016) 24(6):1900–6. 10.1007/s00167-014-3149-4 24990663

[8] Cummins CA Messer TM Nuber GW. Suprascapular nerve entrapment. J Bone Joint Surg Am. (2000) 82(3):415–24. 10.2106/00004623-200003000-00013 10724234

[9] Al-Redouan A Holding K Kachlik D. “Suprascapular canal”: anatomical and topographical description and its clinical implication in entrapment syndrome. Ann Anat. (2021) 233:151593. 10.1016/j.aanat.2020.151593 32898658

[10] Park J Su MY Kim YU. Accuracy of suprascapular notch cross-sectional area by MRI in the diagnosis of suprascapular nerve entrapment syndrome: a retrospective pilot study. Korean J Anesthesiol. (2022) 75(6):496–501. 10.4097/kja.22153 35700981 PMC9726457

[11] Ashton F Swaile H Tambe A. Suprascapular nerve entrapment: current concepts and recent advances. Indian J Orthop. (2025) 59(6):756–67. 10.1007/s43465-024-01302-4 40511344 PMC12151983

[12] Tirman PF Feller JF Janzen DL Peterfy CG Bergman AG. Association of glenoid labral cysts with labral tears and glenohumeral instability: radiologic findings and clinical significance. Radiology. (1994) 190(3):653–8. 10.1148/radiology.190.3.8115605 8115605

[13] Strauss EJ Kingery MT Klein D Manjunath AK. The evaluation and management of suprascapular neuropathy. J Am Acad Orthop Surg. (2020) 28(15):617–27. 10.5435/JAAOS-D-19-00526 32732653

[14] Piasecki DP Romeo AA Bach BR Jr Nicholson GP. Suprascapular neuropathy. J Am Acad Orthop Surg. (2009) 17(11):665–76. 10.5435/00124635-200911000-00001 19880677

[15] Safran MR. Nerve injury about the shoulder in athletes, part 1: suprascapular nerve and axillary nerve. Am J Sports Med. (2004) 32(3):803–19. 10.1177/0363546504264582 15090401

[16] John TS Fishman F Sharkey MS Carter CW. Current concepts review: peripheral neuropathies of the shoulder in the young athlete. Phys Sportsmed. (2020) 48(2):131–41. 10.1080/00913847.2019.1676136 31596162

[17] Chen AL Ong BC Rose DJ. Arthroscopic management of spinoglenoid cysts associated with SLAP lesions and suprascapular neuropathy. Arthroscopy. (2003) 19(6):E15–21. 10.1016/s0749-8063(03)00381-5 12861219

[18] Antoniou J Tae SK Williams GR Bird S Ramsey ML Iannotti JP. Suprascapular neuropathy. Variability in the diagnosis, treatment, and outcome. Clin Orthop Relat Res. (2001) 386:131–8. 10.1097/00003086-200105000-00017 11347826

[19] Brzoska R Laprus H Klaptocz P Malik SS Solecki W Blasiak A. Arm function after arthroscopic decompression of the suprascapular nerve at the spinoglenoid notch and suprascapular notch in volleyball players. Orthop J Sports Med. (2023) 11(2):23259671221147892. 10.1177/23259671221147892 36874055 PMC9974621

[20] Mall NA Hammond JE Lenart BA Enriquez DJ Twigg SL Nicholson GP. Suprascapular nerve entrapment isolated to the spinoglenoid notch: surgical technique and results of open decompression. J Shoulder Elbow Surg. (2013) 22(11):e1–8. 10.1016/j.jse.2013.03.009 23664748

[21] Matsuzawa G Hatta T Asano S Takahashi M Aizawa T. Intraosseous ganglion protruding into the spinoglenoid notch with suprascapular nerve entrapment: a case report. Cureus. (2024) 16(11):e74863. 10.7759/cureus.74863 39737318 PMC11684901

[22] Vij N Fabian I Hansen C Kasabali AJ Urits I Viswanath O. Outcomes after minimally invasive and surgical management of suprascapular nerve entrapment: a systematic review. Orthop Rev (Pavia). (2022) 14(3):37157. 10.52965/001c.37157 35936798 PMC9353691

